# Childhood Maltreatment Is Associated with Larger Left Thalamic Gray Matter Volume in Adolescents with Generalized Anxiety Disorder

**DOI:** 10.1371/journal.pone.0071898

**Published:** 2013-08-12

**Authors:** Mei Liao, Fan Yang, Yan Zhang, Zhong He, Ming Song, Tianzi Jiang, Zexuan Li, Shaojia Lu, Weiwei Wu, Linyan Su, Lingjiang Li

**Affiliations:** 1 Department of Psychiatry, the Second Xiangya Hospital of Central South University, Changsha, China; 2 Department of Radiology, the Second Xiangya Hospital of Central South University, Changsha, China; 3 National Laboratory of Pattern Recognition, Institute of Automation, Chinese Academy of Sciences, Beijing, China; Centre Hospitalier Universitaire Vaudois Lausanne - CHUV, UNIL, Switzerland

## Abstract

**Background:**

Generalized anxiety disorder (GAD) is a common anxiety disorder that usually begins in adolescence. Childhood maltreatment is highly prevalent and increases the possibility for developing a variety of mental disorders including anxiety disorders. An earlier age at onset of GAD is significantly related to maltreatment in childhood. Exploring the underpinnings of the relationship between childhood maltreatment and adolescent onset GAD would be helpful in identifying the potential risk markers of this condition.

**Methods:**

Twenty-six adolescents with GAD and 25 healthy controls participated in this study. A childhood trauma questionnaire (CTQ) was introduced to assess childhood maltreatment. All subjects underwent high-resolution structural magnetic resonance scans. Voxel-based morphometry (VBM) was used to investigate gray matter alterations.

**Results:**

Significantly larger gray matter volumes of the right putamen were observed in GAD patients compared to healthy controls. In addition, a significant diagnosis-by-maltreatment interaction effect for the left thalamic gray matter volume was revealed, as shown by larger volumes of the left thalamic gray matter in GAD patients with childhood maltreatment compared with GAD patients without childhood maltreatment as well as with healthy controls with/without childhood maltreatment. A significant positive association between childhood maltreatment and left thalamic gray matter volume was only seen in GAD patients.

**Conclusions:**

These findings revealed an increased volume in the subcortical regions in adolescent GAD, and the alterations in the left thalamus might be involved in the association between childhood maltreatment and the occurrence of GAD.

## Introduction

Generalized anxiety disorder (GAD) is a common anxiety disorder that usually begins in adolescence, and it affects about 5.7% people in the general population [1]. GAD often co-occurs with major depressive disorder [2] and causes significant distress or impairment in life [3]. However, GAD is less studied compared to other anxiety disorders [4], despite its high prevalence and clinical importance.

The core feature of GAD is pathological anxiety, which is believed to arise from abnormalities in cortical/subcortical interactions based on fear conditioning framework [5], [6], [7]. Sensory fibers from multiple sensory modalities arrive at the amygdala passing through the thalamus [6]. The amygdala integrates different information and induces autonomic and behavioral fear response [8]. The thalamus plays an important role in filtering sensory information and emotional regulation [9]. The insular cortex seems to be associated with modulating subjective feeling states and interoceptive awareness [10]. The prefrontal cortex is involved in emotional regulation by down-regulating the activity of the amygdala and related limbic structures [11]. The medial prefrontal cortex and hippocampus are involved in the process of learning and remembering threat stimulus [5], [6]. Deficits in any of these brain regions or connections between these brain regions might result in pathological anxiety [5], [6], [7], [11].

Because of the less study on GAD, the model for the neural circuitry of GAD is extrapolated from findings in other anxiety disorders, with limited empirical data available. Although a few structural neuroimaging studies have been performed in adolescents with GAD, the results are inconsistent. De Bellis et al. [12] observed an increased amygdala volume in GAD patients compared to healthy subjects, whereas another study [13] found a reduced left amygdala volume in adolescents with different anxiety disorders compared to healthy subjects, and a more pronounced decreased amygdala volume in GAD patients as opposed to those with other anxiety disorders. In addition, two studies in adult GAD patients showed larger amygdala [14], [15] and dorsomedial prefrontal cortex [15] in GAD patients relative to healthy subjects. Given the current limited and inconsistent structural neuroimaging data from GAD patients, the first purpose of the present study was to explore alterations of gray matter volumes in adolescent GAD patients.

Childhood maltreatment is highly prevalent with estimations of more than 30% of the adult population having experienced at least one form of maltreatment during childhood [16], and it increases the possibility for developing a variety of mental disorders including anxiety disorders [17]. Maltreatment includes physical, emotional and sexual abuse, as well as physical and emotional neglect [18]. Epidemiological studies have shown that 40% individuals having experienced childhood maltreatment, whether retrospectively or prospectively ascertained, develop anxiety disorders [17]. An earlier age at onset of GAD is significantly related to maltreatment in childhood [19]. Hence, exploring the underpinnings of the relationship between childhood maltreatment and adolescent onset GAD would be helpful in identifying the potential risk markers for this disease.

Recently, more and more studies have focused on the neurobiological consequences of childhood maltreatment. In animal studies, early adverse experiences, such as maternal separation or abuse, induce a series of long-term alterations on cognitive and emotional regulation, hypothalamus-pituitary-adrenal axis function, and brain morphology [20]. Alterations of brain structure, including decreased dendritic spine density, delayed maturation of neurons, altered neuronal structure and synapse formation, and reduced neurogenesis, have been found in the hippocampus, amygdala and prefrontal cortex [21], [22]. Significant morphological microglial activation has been observed in the thalamus and hippocampus in the rodent after stress [23]. In human studies, neuroimaging techniques have been widely used to investigate the changes of brain structure. In healthy subjects [24] or participants regardless of diagnosis [25], [26], [27], childhood maltreatment is frequently associated with reduced gray matter volumes in the hippocampus [24], [25], [26] and prefrontal cortex [24], [26], [27]. A meta-analysis [28] exhibited that amygdala volume in subjects with maltreatment-related posttraumatic stress disorder did not differ from that in healthy controls. However, recently two studies [29], [30] have reported an increased amygdala volume, whereas one study [31] have found a decreased amgdala volume in healthy adolescents who had experienced childhood maltreatment. Besides, reduced gray matter volumes in the insular [24] and thalamus [32], as well as increased gray matter volumes in the superior temporal gyrus [33] have been reported in healthy samples with childhood abuse. Most brain regions mentioned above are involved in anxiety circuitry, and these maltreatment-related gray matter volume changes were investigated in healthy subjects or participants regardless of diagnosis. Why some subjects having experienced childhood maltreatment eventually develop into GAD, but not the other? Is there any childhood maltreatment related brain structure alteration associated with the occurrence of GAD? The second purpose of the present study, therefore, was to investigate the possible alterations of gray matter volume involved in the association between childhood maltreatment and GAD.

## Materials and Methods

### Subjects

Twenty-six patients with GAD (14/12, with/without childhood maltreatment) and 25 healthy controls (HCs) (12/13, with/without childhood maltreatment), were enrolled in the present study. All subjects were recruited from local high schools in Hunan Province via advertisements and school notices from Oct. 2011 to Jul. 2012. First, 1885 subjects finished the 41-item self-report questionnaire, the Screen for Child Anxiety Related Emotional Disorders (SCARED) [34], [35]. The SCARED is a reliable and valid screening tool for childhood anxiety disorders, with an optimal total cutoff point score of 25 to separate children with anxiety disorders from those without [34], [35]. Then, 508 subjects with positive SCARED scores and 165 in 1377 subjects with negative SCARED scores were diagnosed with DSM-IV criteria and the Schedule for Affective Disorders and Schizophrenia for School Age Children-Present and Lifetime (K-SADS-PL) version [36] by the same clinician. The K-SADS-PL is a semi-structured instrument to ascertain present and lifetime history of psychiatric disorders. In this study the age range of subjects was 16 to 18, so we only interviewed the adolescent. Inclusion criteria for patients in this study were current first-episode, medication-naive, generalized anxiety disorder without co-morbidity disorders. HCs met criteria for no mental disorders or physical diseases and were selected to match GAD patients on age, gender, and childhood maltreatment. Exclusion criteria for all subjects included current major depression disorder, other anxiety disorders, Tourette’s syndrome, conduct disorder, suicidal ideation, lifetime mania, psychosis, or pervasive developmental disorders, mental retardation, any neurological abnormalities, history of seizures, head trauma or unconsciousness, and use of psychoactive substances. All subjects enrolled in this study were medication-naïve, right-handed, and volunteered to participate in this study. Written informed consent was obtained from each adolescent and one of his or her legal guardians after the study had been fully explained. This study was approved by the Ethics Committee of the Second Xiangya Hospital of Central South University, China. Psychological counselors who are responsible for the mental health of the adolescent in these high schools were present in the study. We were asked to give a global evaluation and necessary advices to all participants, including participants who had finished the SCARED but declined to MRI scans and participants who did not meet the inclusion criteria of this study. All potential participants who declined to participate were not disadvantaged in any other way by not participating in the study.

### Clinical Assessment

The Childhood Trauma Questionnaire (CTQ) [37], a 28-item retrospective self-report questionnaire with a total sum score between a minimum of 25 and a maximum of 125, was administered to assess childhood maltreatment in all subjects. The five CTQ subscales respectively assess five kinds of childhood maltreatment, including physical abuse, sexual abuse, emotional abuse, physical neglect, and emotional neglect [37]. Childhood maltreatment (CM) was defined as a “moderate to severe” score on any of five subscales. Moderate-severe cutoff scores for each subscale are ≥ 13 for emotional abuse; ≥ 10 for physical abuse or neglect; ≥ 15 for emotional neglect; ≥ 8 for sexual abuse [38]. The rest of the subjects were considered to be subjects without childhood maltreatment (WCM) according to the CTQ. Additionally, all the patients were assessed with the Penn State Worry Questionnaire (PSWQ) [39] and the Beck Depression Inventory (BDI) [40]. The two questionnaires were introduced to assess anxiety and depression levels in adolescent GAD patients. Table 1 contains the demographic and clinical measures between GAD patients and HCs with nested with or without childhood maltreatment comparisons.

**Table 1:** Demographic, Questionnaire data of adolescent GAD patients and healthy controls.

	GAD (26)		HCs (25)		Statistical value	*p*
	CM(14)	WCM(12)	CM(12)	WCM(13)		
Age (year)	17.0±0.20	16.67±0.22	16.58±0.22	16.85±0.21	*F*_1,47 = _0.308	0.582
Sex	7F/7M	6F/6M	6F/6M	6F/7M	χ^2 = ^0.020	0.886
CTQ	46.79±1.35	32.08±1.46	45.00±1.46	32.70±1.40	*F*_1,47 = _0.173	0.680
Emotional Abuse	9.14±2.32	5.67±0.99	7.58±2.84	6.92±1.55	*F*_1,47 = _0.069	0.794
Emotional Neglect	13.64±2.79	7.83±2.04	12.42±3.32	8.31±1.65	*F*_1,47 = _0.281	0.599
Physical Abuse	6.29±2.43	5.25±0.87	6.00±1.21	5.54±0.97	*F*_1,47 = _0.000	0.997
Physical Neglect	11.00±1.88	7.25±1.36	10.42±2.28	7.92±1.89	*F*_1,47 = _0.009	0.927
Sexual Abuse	6.71±1.82	5.08±0.29	5.75±1.49	5.15±0.38	*F*_1,47 = _1.710	0.197
BDI	9.54±4.61	8.67±4.48	–	–	t = 0.479	0.637
PSWQ	52.5±9.08	57.83±8.64	–	–	t = −1.526	0.140

Means and standard deviations (±) are given.

GAD, generalized anxiety disorder; HCs, healthy controls; CTQ, childhood trauma questionnaire; BDI, the Beck Depression Inventory; PSWQ, the Penn State Worry Questionnaire; CM, childhood maltreatment; WCM, without childhood maltreatment.

### Structural Magnetic Resonance Imaging (MRI) Acquisition

MRI examinations were conducted at the Second Xiangya Hospital of Central South University, in China and performed with a Philips 3.0 Tesla Scanner, equipped with a SENSE-8 channel head coil. For each participant, T1-weighted high-resolution anatomical images were obtained using a 3-dimensional (3D) rapid acquisition gradient echo sequence, repetition time (TR) = 7.5 milliseconds, echo time (TE) = 3.7 milliseconds, flip angle = 8°, field of view = 256 mm×256 mm, slice = 180, voxel size = 1 mm×1 mm×1 mm.

### Image Analysis

Image analysis was conducted with SPM8 (http://www.fil.ion.ucl.ac.uk/spm/) and the VBM8 toolbox (VBM8, version 435; http://dbm.neuro.uni-jena.de/vbm8/). Individual structural images were preprocessed with the VBM8 toolbox following the default parameter. T1-weighted images were corrected for bias-field inhomogeneities, spatially normalized to the Montreal Neurological Institute standard template space, and segmented into gray matter, white matter and cerebrospinal fluid, within a unified model [41] including high-dimensional DARTEL normalization. Gray matter segments were modulated by the non-linear components only, which allows comparing the absolute amount of tissue corrected for individual brain sizes. The voxel resolution after normalization was 1.5 mm×1.5 mm×1.5 mm. The check data quality function was adopted to check homogeneity of gray matter images. No inconsistencies were found among these gray matter images. Finally, the segmented, modulated gray matter images were smoothed by a Gaussian kernel of 8 mm FWHM.

### Statistical Methods

Statistical analysis for the demographic and clinical measures was performed by means of a general linear model with a 2 (diagnosis: GAD vs HCs)×2 (childhood maltreatment: CM vs WCM) comparison, chi-square test or *t* test, as needed, in SPSS16.

Image statistics were conducted with second-level models in SPM8. The smoothed gray matter images were entered into a voxel-by-voxel general linear model with a 2 (diagnosis: GAD vs HCs)×2 (childhood maltreatment: CM vs WCM) comparison, controlling for age and gender, to assess the diagnosis main effect (GAD > or < HCs), the maltreatment main effect (CM > or < WCM), and the diagnosis-by-maltreatment interaction effects. According to the aim of this study, the diagnosis main effect and the diagnosis-by-maltreatment interaction effects were of particular interest. We defined the amygdala, thalamus, insula, hippocampus and prefrontal cortex (especially the medial prefrontal cortex) as our regions of interest (ROIs), given their important roles in anxiety circuitry. The ROIs were defined according to Tzourio-Mazoyer et al. [42] and the ROIs masks were created by means of the WFU PickAtlas [43]. Then a supplementary whole brain analyses was conducted to examine non-hypothesized regions. For ROIs and whole brain analysis, a family wise error (FWE) rate correction for multiple comparisons was used with a threshold of *p* < .05. For the brain region of significant diagnosis-by-maltreatment interaction effect, the mean contrast values were extracted from each subject and further analyzed with SPSS16. We conducted a general linear model analysis and simple effect analysis to show the diagnosis-by-maltreatment interaction effect.

To supplement the interaction effect, we further conducted a whole brain regression analysis in SPM8 to investigate the relationship between childhood maltreatment and regional gray matter volume by regressing CTQ scores on the gray matter volume images in the separated groups, as well as in the combined group. A family wise error (FWE) rate correction for multiple comparisons was also used with a threshold of *p* < .05.

## Results

### Demographic and Clinical Measures

The results are listed in Table 1. There were no significant differences between the groups in age, gender, CTQ scores, and subscales of the CTQ. No significant differences were found in BDI and PSWQ scores between adolescent GAD patients with or without childhood maltreatment.

### Structural Alterations in Gray Matter Volumes

**Figure 1:**
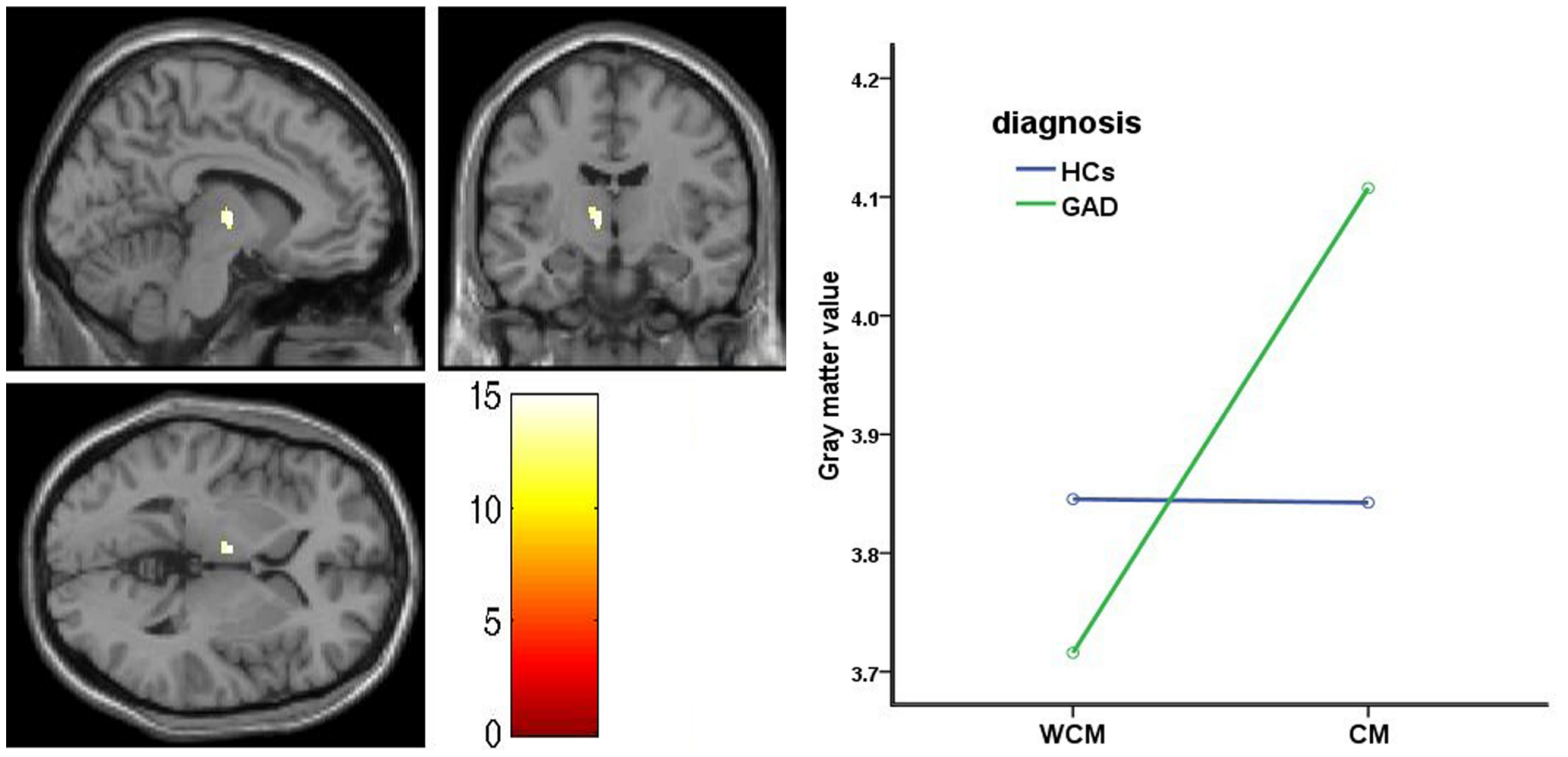
The diagnosis-by-maltreatment interaction effect in the left thalamus. Left: Statistical parametric map depicting interaction effect in the left thalamus (*p* < 0.05, FWE corrected). Color scales represent F-values. Right: The interaction graph showing left thalamic gray matter volume differences between the groups, in which adolescent GAD patients with childhood maltreatment have more gray matter volumes in the left thalamus than those without childhood maltreatment and both HCs. GAD, generalized anxiety disorder, HCs, healthy controls, CM, childhood maltreatment, WCM, without childhood maltreatment.

We found no diagnosis or maltreatment main effects in all ROIs, controlling for age and gender. However, a significant diagnosis-by-maltreatment interaction effect was observed in the left thalamus (*F*_1,45 = _14.96; *p = *0.031, FWE corrected; x = −8, y = −10, z = 1, cluster size = 83 voxels), as shown in Figure 1. No other ROIs showed significant interaction effect.

**Figure 2:**
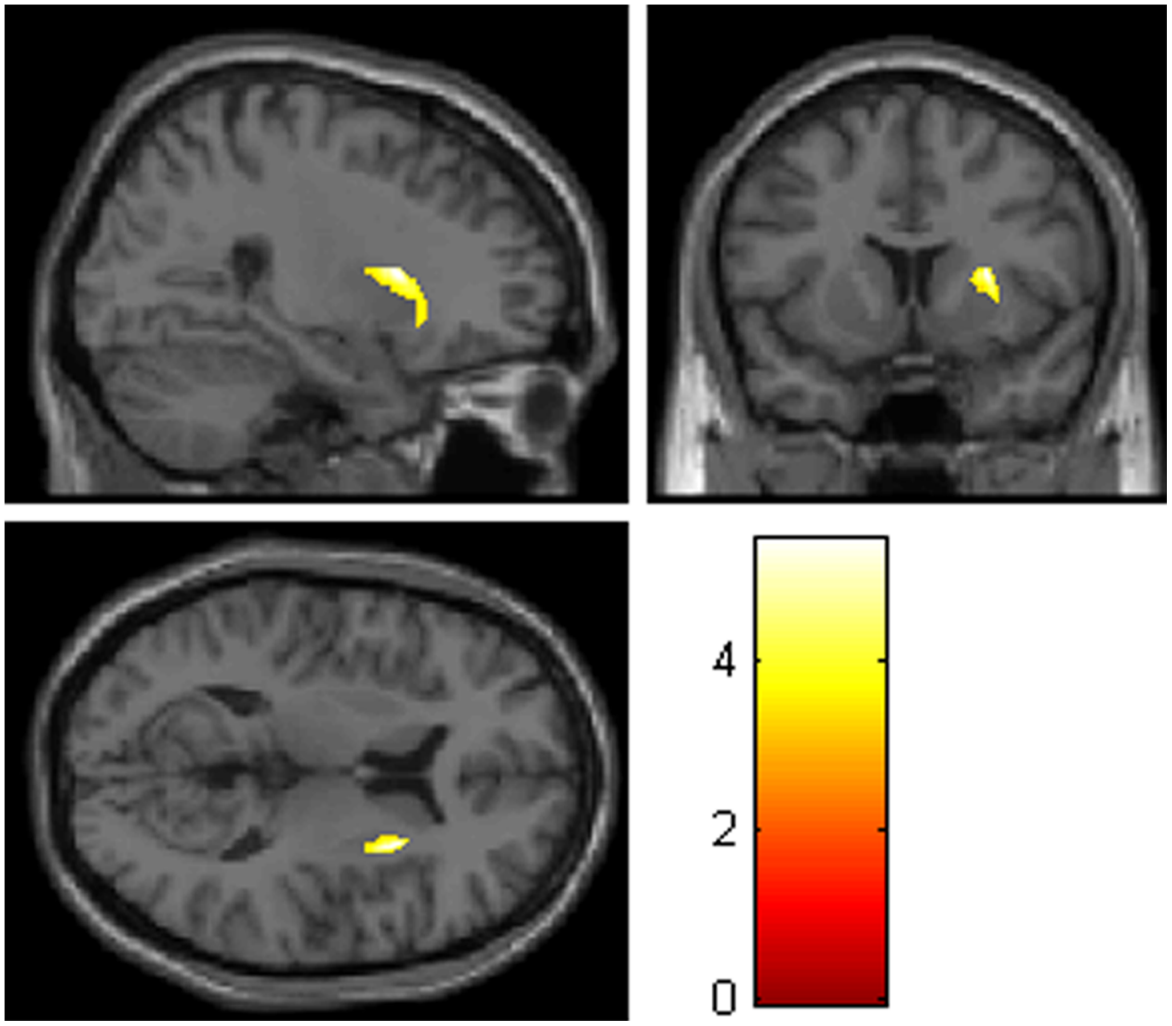
Increased right putaminal gray matter volume in adolescent GAD patients compared to healthy controls (*p* < 0.05, FWE corrected). Color scales represent t-values.

The whole brain analysis revealed a significant diagnosis main effect in the right putamen (*F*_1,45 = _29.51; *p = *0.044, FWE corrected; x = 27, y = 11, z = 10, cluster size = 263 voxels), with larger gray matter volume of the right putamen in adolescent GAD patients compared to healthy controls (GAD > HCs; Figure 2). No maltreatment main effect was found in the whole brain analysis controlling for age and gender. Whole brain analysis found no other brain regions except the left thalamus exhibited significant interaction effect.

Since a significant diagnosis-by-maltreatment interaction effect was observed in the left thalamus, the mean contrast values of this brain region were extracted. The general linear model analysis in SPSS, controlling for age and gender, also showed a significant diagnosis-by-maltreatment interaction effect in the left thalamus, that was adolescent GAD patients with childhood maltreatment had significantly larger gray matter volumes than adolescent GAD patients without childhood maltreatment and both HCs in the left thalamus (diagnosis-by-maltreatment; *F*_1,45 = _5.440, *p* = 0.024) (Figure 1). We compared subjects with childhood maltreatment and those without childhood maltreatment on each diagnosis level. The results exhibited that the maltreatment-related alteration in the left thalamus was only observed in adolescents with GAD (t = −3.514, *p* = .002), but not in HCs (*p* > .05).

**Figure 3:**
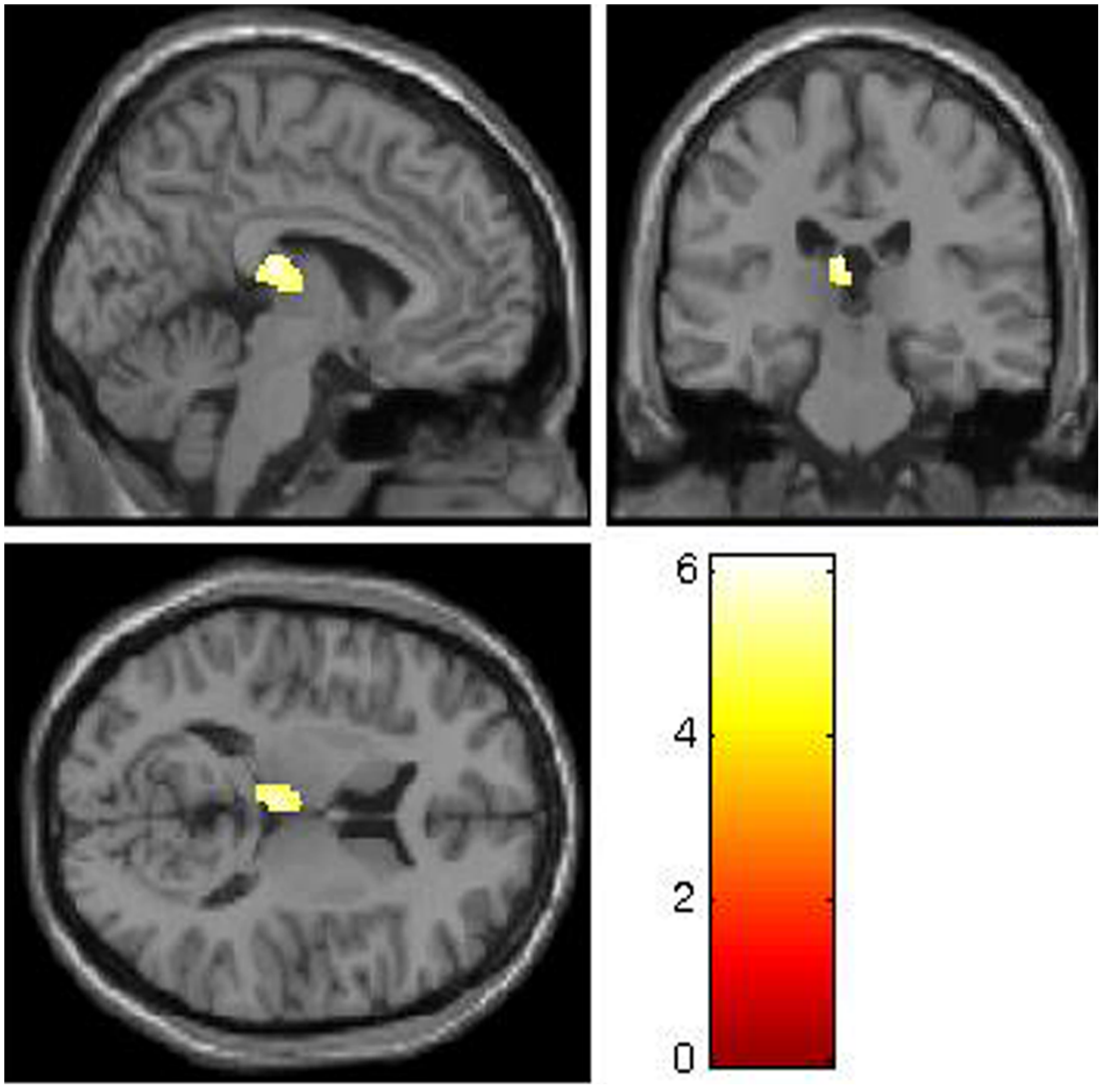
Statistical parametric map depicting the positive association between childhood maltreatment (Childhood Trauma Questionnaire [CTQ] scores) and left thalamic gray matter volume (mean contrast values) in GAD patients (*p* < 0.05, FWE corrected). Color scales represent t-values.

The regression analysis only yielded a strong positive association between CTQ scores and left thalamic gray matter volume in adolescent GAD patients (x = −5, y = −24, z = 15; *t* = 6.16, df = 24; *p*_FWE-corrected = _0.036, cluster size = 237), as shown in Figure 3. No brain regions were found to be significantly associated with CTQ scores in HCs or combined group.

## Discussion

We employed high-resolution structural magnetic resonance imaging and voxel-based morphometry approaches to study alterations in gray matter volume, as well as the association between childhood maltreatment and alterations in gray matter volume in adolescent GAD patients in the current study. The results of the present study showed larger gray matter volume in the right putamen in adolescent GAD patients and a diagnosis-by-maltreatment interaction effect in the left thalamus. Further analysis exhibited the significant maltreatment-related alteration in the left thalamus was only found in adolescents with GAD, but not in HCs.

The finding that exhibited no alterations in prior-set ROIs but larger gray matter volumes in an unexpected brain region, the putamen, in GAD subjects seems interesting. To the best of our knowledge, this is the first investigation in GAD reporting putaminal gray matter alterations. The putamen, which belongs to the basal ganglia, has widely spread functional connections with cortical and subcortical areas in the brain [44]. The putamen has been suggested to be related to a number of anxiety disorders and anxiety symptoms, such as GAD [45], social anxiety disorder [45], posttraumatic stress disorder [46], panic disorder [47], obsessive-compulsive disorder [48], lactated-induced anxiety [49] and anxiety symptoms in Parkinson disease [50]. Besides, GAD patients often accompany with somatic symptoms which are associated with sympathetic dysregulation [51]. Previous researches suggested several adrenergic receptors and dopamine receptors exerting an important influence on sympathetic regulation exist in the putamen [52], [53] . Therefore, alterations in the putamen might be associated with somatic anxiety symptoms of GAD.

The amygdala plays an important role in processing emotional valence and generating rapid fear response [5], [6], [8]. Several studies on GAD have reported alterations of gray matter volumes in the amygdala. Two studies in adult GAD patients exhibited larger amygdala [14], [15], whereas the other two studies in adolescent GAD patients yield inconsistent results in the amygdala [12], [13]. Three of these studies investigated GAD patients with co-morbid diagnosis [12], [13], [14], while GAD patients in this study had no co-morbidity disorders. This might partly explain the different findings on the amygada. Although one previous study also examined GAD patients without co-morbid diagnosis, all the subjects were female. Gender differences on amygdala gray matter volume have been reported in many researches [54], [55], [56]. In addition, as we described earlier, childhood maltreatment has been suggested to be associated with alterations of the amygdala volume [29], [30], [31]. All factors mentioned above might complicate the results on alteration of the amygdala.

This is the first study to investigate the possible association between childhood maltreatment and gray matter volumes in GAD patients. Epidemiological evidences have shown that childhood maltreatment would increase the risk of GAD [17], [18]. Our finding suggested a diagnosis-by-maltreatment interaction effect in the left thalamus and revealed a strong positive association between childhood maltreatment and left thalamic gray matter volume only in GAD patients. It partially suggested that the left thalamus might be the childhood maltreatment related brain structure that would increase the risk of GAD. The diagnosis-by-maltreatment interaction effect in the left thalamus might be the reason why some subjects with childhood maltreatment develop into GAD but not the others. The thalamus, a major relay center of the brain with strong reciprocal connections with cortical and subcortical structures, such as the prefrontal cortex and amygdala, is a critical component of the cortical-(amygdalo)-thalamic circuits which plays a crucial role, not only in filtering sensory information, but also in higher cognitive functions and emotional regulation [6], [7], [9], [57]. Changes in the thalamus, which is implicated in sensory information filtering and alertness [9], [57], [58], might induce pathological anxiety. Chronic stress increases the state of alertness [59], which is associated with the thalamus [58]. Consistent with our result, a Positron Emission Tomography study [60] revealed significantly greater regional cerebral glucose metabolism in thalamus in adult monkeys who experienced early life stress compared to controls, and another study [61] showed that young adults who experienced corporal punishment in childhood exhibited increased cerebral blood volume in the thalamus. Greater activation or increased volumes in the thalamus might suggest a general problem with sensory information processing, perhaps indicating hyper-vigilance, which is thought to be involved in the pathophysiology of GAD. Structural and functional alterations in the thalamus might reflect a maltreatment-related increase in sensitivity to conditional sensory information in the environment.

However, one previous study [32] compared 31 physically abused children and 41 non-abused children regardless of mental disorders, and found reduced bilateral thalamic gray matter volumes. In our study, the more reported forms of maltreatment were physical and emotional neglect. It is possible that different forms of childhood maltreatment might be associated with different alteration patterns of the thalamic gray matter volume. In addition, the study conducted by Hanson et al. [32] investigated the possible linking between physical abuse and neurophysiological alterations in a general population regardless of mental disorders, whereas we focused on a possible association between childhood maltreatment and brain deficits in GAD patients. The heterogeneity of the sample might also account for the inconsistent results.

Brain regions including the hippocampus [24], [25], [26] and prefrontal cortex [24], [26], [27] have been frequently reported to be associated with childhood maltreatment. Preclinical studies have confirmed that early adverse experiences induce alterations of the hypothalamus-pituitary-adrenal axis functions and further result in stress-related changes on the hippocampus [21] and prefrontal cortex [22].We did not find any significant association between childhood maltreatment and the hippocampus and prefrontal cortex at a harsh statistical threshold in this study. However, we found a negative association between childhood maltreatment and left prefrontal gray matter volume in GAD patients and combined group, at a more lenient threshold of *p* < .001, uncorrected. The maltreatment-related alteration in the prefrontal cortex is consistent with previous findings [24], [26], [27]. As concluded in a review, prolonged stress exposure causes architectural changes in prefrontal dendrites [22]. The prefrontal cortex is critically implicated in emotion regulation processes by down-regulating the limbic structures [5], [6], [11]. Volume reduction in the prefrontal cortex could be associated with insufficiencies in emotion regulation and therefore increase the vulnerability for pathological anxiety [22]. The hippocampus did not show any association with childhood maltreatment in any group even at a lenient threshold of p < .001, uncorrected. A possible explanation for this phenomenon is delayed effects of early stress on hippocampal development [62]. Reduced hippocampal volume has been consistently reported in adults with histories of childhood maltreatment, but this change has been rarely found in children with childhood maltreatment [28]. Animal studies also suggested that effects of early life stress on hippocampal morphology do not become apparent until adulthood [62].

The findings in the present study showed lateralization, such as larger right putaminal gray matter volume, and a positive association between childhood maltreatment and left thalamic gray matter volume in GAD patients. The reason for such lateralization might be the cases that findings on one side exceed the statistical threshold, while results on the other side did not. In this study, a positive association between childhood maltreatment and right thalamic gray matter volume was found at a more lenient threshold of *p* < .005, uncorrected. This association was not apparent at a more rigorous statistical threshold. However, even at a more lenient threshold of *p* < .005, uncorrected, no difference was found in left putaminal gray matter volume between GAD patients and HCs. The lateralization to the right is consistent with valence lateralization hypothesis, which suggests the right hemisphere is dominant for negative emotions [63]. The lateralization phenomena revealed in this study needs to be further clarified in the future study.

Some limitations of the current study must be acknowledged. First, the sample in this study was relatively small and we only studied first-episode, medication-naive, adolescent GAD patients aged 16 to 18, which might limit the generalizability of our findings. Second, the childhood trauma questionnaire introduced to assess childhood maltreatment is a retrospective self-report questionnaire, which could result in a recall bias. Although one epidemiological study [17] found no differences between prospective and retrospective reports in predicting associations between childhood maltreatment and adult psychopathology, patients with GAD could have a more negative recall bias and a better memory of childhood maltreatment. Third, this is a cross-sectional study, which cannot explain the direct relationships between childhood maltreatment and the occurrence of GAD. Forth, image pre-processing steps such as registration and smoothing in voxel-based morphometry might lead to different results [64].

In conclusion, we reported an increased gray matter volume of the right putamen in subjects with GAD relative to HCs, and a strong positive association between childhood maltreatment and left thalamic gray matter volume only in GAD patients. The increased gray matter volume of the right putamen suggests that pathological change of the putamen may be one of the neural substrates underlying the occurrence of GAD. The thalamus might be involved in the association between childhood maltreatment and the occurrence of GAD. In future studies, the impact of childhood maltreatment should be noted. Since childhood maltreatment is closely associated with GAD and increases the risk of this disorder by modulating brain structures, it seems that neuroimaging studies have been confounded by those multiple effects caused by childhood maltreatment. It should also be noted that there are sensitive periods during which specific brain regions are vulnerable to early adversity [26], and childhood maltreatment-related brain structural alterations might occur at a specific age. Anyway, a longitudinal investigation with a large sample is required to validate the results in our study.
